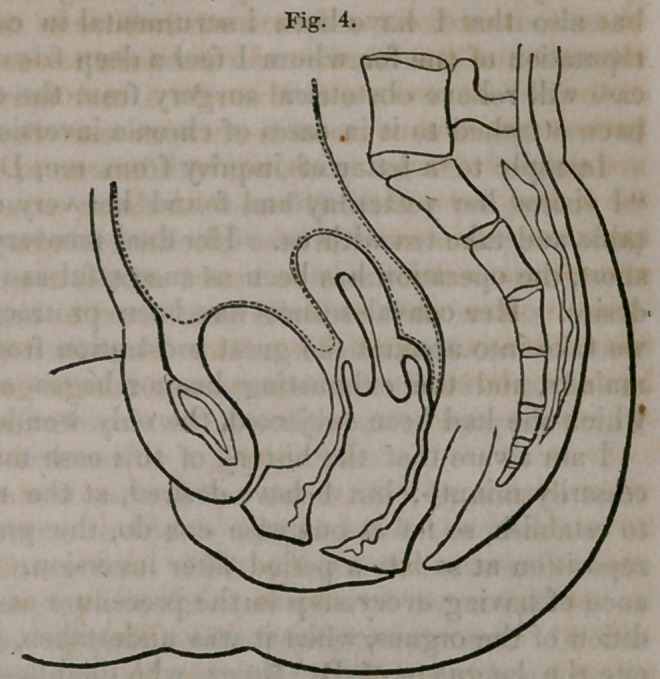# Report of a Case of Inversion of the Uterus Successfully Reduced after Six Months

**Published:** 1858-09

**Authors:** 


### ECLECTIC DEPARTMENT,

### AND SPIRIT OF THE MEDICAL PERIODICAL PRESS.

---

*Report of a Case of Inversion of the Uterus Successfully Reduced after Six Months, with Remarks on Reduction in Chronic Inversion.* By James P. White, M. D., Professor of Obstetrics in the University of Buffalo, and Accoucheur to the Buffalo Lying-in Hospital. (With four wood cuts.)

The following report of a case of inverted uterus was made at a meeting of the Buffalo Medical Association, February 12th, 1856, and appears in its published proceedings:

"On Monday, 28th January, Dr. Storck called at my office requesting my attendance, with himself and Dr. Dupré, upon a young female at No. 9 Huron street, who had been delivered of her first child upon the Tuesday previous.

"Accompanying him, I found the patient, 19 years old, exsanguine, with quick pulse, and greatly exhausted from loss of blood. I found that she had been attended, at the time of her delivery, by a German midwife, who stated that, after a brief labor, she had given birth to a male infant, weighing ten and one-half pounds. She also stated that the after-birth soon came away, accompanied by a large tumor, which descended into the vagina. This tumor she supposed to be a mole or false conception, and she stated that it was as 'large as a cannon-ball.' The flooding, at the time, she described as terrific, producing protracted syncope.

"A day or two previous to my first visit, whilst making an effort to evacuate the bowels, the tumor had descended through the os externum and became suspended between the patient's thighs. All her efforts to remove the tumor proving unavailing, the midwife had sent for the gentlemen above mentioned for that purpose, and they now associated me with them in the treatment of the case.

"The tumor was immediately recognized as the inverted uterus, as large as at the fourth month of pregnancy, which, with the external organs, was inflamed and tender. The uterus felt hard and inflexible, the body being apparently distended with blood from the ligated condition of the neck, and the parts had been rendered exceeding irritable by manipulations for the removal of the supposed 'false conception or tumor.'

"By grasping the uterus gently and firmly with both hands, which it completely filled, compression was continued until the organ was relieved of its engorgement and considerably diminished in bulk. By continuing this firm but gentle compression, I was at length enabled to carry it up into the vagina. At this time the patient, having lost some blood during the effort, became very faint, in consequence of which, and the sensitiveness of the vulva, it was deemed prudent to omit further efforts at restoration of the organ until the following morning.

"Meanwhile the bowels were moved by an enema, followed by an anodyne, and fomentations were applied to the abdomen and external genitals. The patient was also directed to take freely of broths and stimulants.

"*Tuesday* 29*th*, 12, M. Slept pretty well during the night; the hypogastric and vulval soreness is considerably diminished; the uterus lessened in size, and manifests some susceptibility to indentation upon pressure. The hæmorrhage has continued during the night; pulse 144, and feeble; has had a severe chill, and is greatly prostrated. During the last three hours Dr. Storck has, at my suggestion, applied extract of belladonna to the neck of the uterus. At 12 o'clock, placed the woman across the bed, her pelvis resting upon its edge and her feet being supported, one in the lap of Dr. Dupré, the other in that of Dr. Storck. Prof. S. B. Hunt, who was kind enough to visit her, upon my invitation, gave her chloroform so as gradually to bring her moderately under its influence. This effect was maintained throughout the succeeding operation.

"I now placed myself upon my knees between the limbs of the patient, a position admitting of free motion on my own part, giving complete control of the pelvis of the woman, and which could be maintained for a considerable period without unnecessary fatigue to the operator. Introducing the entire right hand into the vagina, the whole body and fundus of the organ were firmly and continuously compressed for some time. At length, keeping up the pressure, it was found, upon applying the thumb to the fundus, that a slight depression could be made. Having succeeded in dimpling the fundus, pressure was maintained with the thumb at that point until the hand became so fatigued as to be nearly powerless. To preserve this depression whilst the muscles of the hand were permitted to relax, a rectum bougie, about twelve inches in length and one in diameter, was carried along its palm fixed in the dimple, and pressure unintermittingly continued through it by the left hand outside the vulva. So soon as the intra vaginal hand was sufficiently rested, pressure by it was recommenced and the bougie withdrawn.

"Whenever these progressive efforts were resumed, the left hand was placed over the uterine tumor, which could now be distinctly felt in the hypogastrium. By means of the counter pressure above the pubis, a much greater degree of pressure could be made upon the depression in the fundus of the uterus without lacerating its vaginal connections. At length the fingers of the left hand being pressed well down into the abdomen, seemed to fasten upon or hook over the anterior uterine lip and aid in its reflexion over the organ. Thus securely held between the two hands, one within the vagina and the other upon the hypogastrium, these efforts at reduction were continued until I became nearly exhausted from fatigue. Gradually the concavity of the fundus was found to be deeper and deeper, until it finally became completely restored. The bougie was now passed up to the fundus, penetrating twelve or more inches beyond the vulva, and gently maintained there by Prof. Hunt, whilst the patient was placed in bed. My fingers were benumbed and nearly deprived of sensation by the long-continued unremitting pressure, and at my request, he also examined to ascertain whether the organs now occupied their normal relations. This being determined by him affirmatively, the bougie was gently withdrawn, and the patient left with directions that an anodyne be administered, quietude preserved, and stimulants and nourishment given freely. It may be added that she seemed more comfortable than before the operation, and expressed herself as feeling better than since her confinement. The hæmorrhage was, from this moment, completely arrested.

"On the 30th, at 11, A. M., upon visiting her, with the same medical gentlemen who were present the day before, found her feeling better, with less pain and much more hopeful. The pulse had, however, but slightly diminished in frequency (140) or increased in force, and she still looked exsanguine.

"Continued the treatment of the day previous, giving as much beef essence and brandy as the stomach will retain.

"On Thursday, 31st, at 11, A. M., Dr. Storck informs me that the irritability of the stomach, which had been troublesome from her delivery, was now greatly increased, and it was with difficulty that she retained the smallest quantity of fluid. The pulse is more feeble, and she is evidently sinking. Notwithstanding the free use of quinine and brandy, she expired at 5, P. M. on the same evening.

"Feb. 1st, at 12, M., the *post-mortem* examination was made by Dr. Lemon, in the presence of Drs. Storck, Dupré, Hauenstein, and Prof. Hunt, the last of whom, at my request, furnishes the following report of the condition of parts as they were found upon examination:

'The examination was held eighteen hours after death. Only the abdominal cavity was opened. All the tissues were extremely bloodless. The stomach and intestines were fully inflated with gas, but almost without any liquid contents. The walls of the intestines were white and translucent, and no trace of inflammatory injection could be found either upon them or any portion of the peritoneum. There was, however, a little serous effusion within the peritoneum, and between some of the convolutions of the intestines a very little lymph was exuded. The uterus was drawn up and removed with as little of the vaginal canal as could be reached from within. Externally the uterus presented its normal shape and position, there being no trace of its recent inversion. The vaginal mucous membrane and the os uteri presented the dark color usual to the organ at this period after labor. The tissues were not softened, nor was there any laceration of them at any point. Upon section through the posterior wall, the same pale, bloodless appearance, noticed elsewhere, was presented. The uterine cavity contained a little altered blood. Upon washing the surface it presented no unusual appearance. The situation of the placenta was marked by the usual rough, flocculent surface.

'The examination revealed no cause of death, unless the anæmic condition of the tissues may be considered as such. I have never before seen so bloodless a subject, with one exception: that of a girl who died from purpura hæmorrhagica.'

"The uterus and its appendages were then submitted to the association for inspection.

"This case is regarded as interesting in many respects. It will encourage the growing belief among accoucheurs, that reduction may be undertaken, with reasonable hope of success, at a period much later than most writers have heretofore advised. Denman, Dewees, Velpeau and others, believe any effort at restoration useless after a very few hours. In a valuable paper upon this subject from the editor of the *Buffalo Medical Journal*, to be found in the November number, 1853, sixty-seven cases are collected, and all the facts pertaining to their reduction, so far as they could be obtained, are given. Most of the cases which were successfully treated were operated upon very soon after the accident. Thirty-two of the sixty seven were not reduced, and a few 'exceptional cases' at various periods after the first day. By this table Dr. Hunt has shown that treatment has, though very rarely, resulted in success at a later period than was formerly supposed practicable, and the above case furnishes another instance in support of the same position.

"I have witnessed but three cases of inversion of the uterus. The history of one is given above. One of the others was seen and reduced soon after the accident; whilst the third was not visited until the fifteenth day—no effort at reduction being attempted. With my present views upon this subject, I should abandon such a case as hopeless only after a prolonged effort at reposition. The accident occurred in 1842, and the female, then but 19 years old, now enjoys tolerable health, though the uterus still remains inverted in the vagina. The case is referred to, and the course of treatment pursued given in part by Prof. Hunt at page 335, in the paper already cited.

"The position in which the patient was, in the present instance, placed for the operation, is deemed worthy of note. It will be perceived that it gives complete control of the pelvis, permits free motion of the person and arms of the operator, and may be maintained for a long time without fatigue. In this position he is able to render important assistance in the most difficult stage of the operation, with the left hand over the hypogastrium. Nor was the use of the bougie unessential; by its assistance continuous pressure was maintained, whilst the hand was relieved for a short period, thus, as it were, tiring out the circular fibres of the neck. How much of the success of the operation was due to the relaxation of the neck from the application of the belladonna, if, indeed, any beneficial influence was exerted by it, I cannot determine. The moderate anæsthesia, continued during the efforts of manipulation, doubtless saved the patient much pain, and lessened involuntary resistance. Whether the patient's chances of rallying were improved by the reposition, may, by some, be deemed doubtful. There were no lesions of the utero-vaginal connection found, indicating that such a degree of force had been used as to impair the integrity of, or excite inflammation in those tissues. The hæmorrhage, which had been considerable during the previous twenty-four hours, ceased with reduction, and the woman was much more comfortable the day following than the one preceding. The patient doubtless died from loss of blood immediately attending the delivery of the placenta and inversion of the uterus, the disturbance of the system occasioned by the unnatural position of that organ during eight days, and the continuous drain by hæmorrhage during the same period. I believe it to be the opinion of all present, that the shock of the operation was fully compensated for by the increased comfort of the patient and arrest of flooding. She had, however, lost too large an amount of blood; reäction could not be established, though nature was aided in her efforts by all the resources of art."

Fully convinced, from the result of the efforts made in this instance eight days after inversion, of the feasibility of restoring the uterus in many cases heretofore considered irreducible, I did not meet with any opportunity of putting my convictions again to the test until the month of March last. The infrequency of the occurrence of this accident among careful practitioners is apparent from the fact, that many largely engaged in practice pass their whole lives without meeting with a single instance. On this point we may cite the reliable statement of Dr. West, in his work on the *Diseases of Women*, that "in the annals of the Dublin Lying-in Hospital, and those of the London Maternity Charity, it was not once met with in a total of more than 140,000 labors."

On the third of March, Dr. C. D. Robinson, of Hornellsville, wrote to me stating that he "had been called in consultation with a neighboring physician, and found a patient with an inverted uterus of more than five months' standing." My opinion was desired as to "the possibility of returning the inverted organ and the propriety of extirpation." In my reply the impropriety of removal, unless as a last resort, was insisted on, and the hope expressed that a prolonged and well directed effort might succeed in reduction; and that, in my belief, it was due to the poor woman that the attempt should be made before she was abandoned to the evils of chronic inversion. A few days subsequent to this date, I was requested to visit her at my earliest convenience.

Engagements in the city prevented my complying with this request until the twelfth of March. On accompanying Dr. Robinson to the residence of the patient, Mrs. Amelia Miller, I found her extremely anæmic, confined to her bed, and suffering greatly from the loss of blood.

The history of the case, as furnished by herself, her husband, and the medical gentlemen who had been in attendance, is as follows: At the age of 30 she was taken in labor at the maturity of her second pregnancy, on the 22d day of September last, Dr. Batten in attendance. This labor was natural to the conclusion of the second stage, when she gave birth to a large male child. Placenta adherent, but removed at the expiration of about thirty minutes, and its delivery followed by copious flowing, severe pain, and faintings. The prostration was so great as to require the constant use of stimulants during the succeeding forty-eight hours, and for three weeks she continued extremely weak and faint. At about this time she took an aloetic cathartic, which occasioned violent efforts at stool, accompanied by pains resembling those of labor. Profuse hæmorrhage followed these straining efforts and a large pear-shaped tumor made its appearance through the os externum. The neck or smaller extremity of this body was at the vulva, and the larger extremity down between her thighs. By the assistance of her husband, she was enabled to return this tumor within the vulva, when a messenger was dispatched for Dr. Batten. Dr. B., upon his arrival, introduced his hand into the vagina and carried the uterus high up into the pelvis, and resorted to astringents and cold for the purpose of arresting the flow of blood, which continued profuse and difficult to control. The prostration being at this time very great, the horizontal posture was enjoined, stimulants and tonics given, and the bowels moved by enema.

During the succeeding three months she had occasional hæmorrhages, which were severe, with constant discharge of muco-sanguinolent matter. Two or three times during this period she so far improved as to walk about her room and partially supervise her domestic affairs, though looking very pale and being very feeble. About the middle of January she had another severe attack of hæmorrhage, the tumor again presented externally, and was again returned as before; that is, pushed back within the vulva. Dr. B. again visited her, and prescribed such remedies as seemed necessary to control the flowing. Since about the first of February she has been compelled, from the debility consequent upon the exhausting sanguinolent and leucorrhœal discharges, to preserve the recumbent posture. Lactation, doubtless, added to the exhaustion, and being confined to her bed she had little appetite, the stomach was irritable and the bowels costive. Ever since the patient took the aloetic cathartic and the tumor made its first appearance between the thighs, she has been aware of the existence of something unnatural in the vagina. This body has occasionally made its appearance externally, requiring the assistance of her husband to replace it, and she has had frequent attacks of a "straining sensation" described as accompanying its first complete descent. She has suffered greatly from all the symptoms arising from exhaustion and sympathy with the uterine irritation decessarily developed by its malposition. The pulse now numbers 130; she is blanched or wax-colored, and grows dizzy and faint when raised to the semi-recumbent posture, and cannot be moved without producing a sense of prostration. It should have been stated, that on the 25th of February Dr. Robinson, of Hornellsville, and Dr. Reynale, of Downsville, visited the patient in consultation with Dr. Batten, when inversion of the uterus was diagnosed, and measures resorted to, calculated to ameliorate her condition.

Fig. 1.

Upon making a careful examination (nearly twenty-five weeks having now elapsed since confinement,) the fundus of the uterus is found just within the os externum, and about one inch and three-fourths in its transverse diameter, and scarcely exceeding an inch in its antero-posterior diameter, Fig. 1. The body and neck of the organ occupy the vagina, and the neck is not more than one inch in diameter, and feeling like the pedicle of a polypus. The inversion is recognized as complete, and the organ scarcely, if at all, larger than when in its natural position six months after delivery. Introducing a large cylindrical speculum into the vagina, the fundus of the uterus passes readily into its cavity, thus demonstrating the complete involution of the uterus, and bringing distinctly into view the rough mucous membrane of its now outer covering, which bleeds upon the slightest touch with the finger or sound. It is seen to be covered with muco-purulent matter also, and not susceptible of indentation by pressure with the point of the sound.

The bowels having been freely moved by an enema, I proceeded to the operation of reduction in the presence of Drs. Robinson, Reynale, Batten,

Dimick, and Mr. J. W. Robinson, medical student. The patient was placed for the operation, as before, upon an elevated, firm bed, with the hips brought quite to its edge, the knees separated, the feet resting in the laps of Drs. Reynale and Robinson, with directions to each to support a knee and hand of the patient, and prevent her from moving about. Dr. Batten brought the patient moderately under the influence of chloroform, which was continued throughout the operation, whilst I placed myself upon my knees, between the limbs of the patient, her pelvis being at a convenient elevation for manipulation. I introduced my right hand completely into the vagina, and firmly grasped the entire body and neck of the uterus. It may here be remarked that the parts were so firmly contracted as to render the introduction of the hand difficult. At the same time that the entire uterine tumor was grasped by the right hand, the large rectum bougie described in the first operation, was carried up, and also received into its palm, and held firmly in contact with the fundus of the uterus, the hand being sufficiently large to receive both, and keep them in apposition. Continuous, gentle pressure was now made upon the external extremity of the bougie with the left hand, whilst the right compressed the uterine tumor, and kept the upper extremity of the instrument directly upon the fundus, and with the dorsum of the hand in the concavity of the sacrum, directed the force in the axis of the pelvic cavity, putting the vagina completely upon the stretch, Fig. 2. This pressure was exerted, and this position unintermittingly maintained, although the force was not to such a degree as to endanger laceration of the utero-vaginal connection, until my strength was nearly exhausted from continuity of effort. At length, and when about to relinquish the task, the uterine tumor began to shorten *at its neck*, and the mouth of the organ to push upon the upper surface of the hand. No depression or dimpling of the fundus was at any time perceptible.—Ascending more and more rapidly as the neck diminished in length, Fig. 3, the fundus finally

Fig. 2.

Fig. 3.

passed out of the hand, and was easily pushed by the bougie through the mouth and neck of the organ up to its proper position, Fig. 4.

Fig. 4.

In order to verify the restoration, Simpson's sound was introduced alongside of the bougie, and was found to enter a little more than two and one-half inches above the os, which could now be distinctly felt. The large speculum, already referred to, was now slipped up around the bougie, when the os was brought distinctly into view, surrounding also the bougie. The sound was again carried through the os to the fundus, through the speculum, and all the medical gentlemen present saw that it passed easily beyond the mouth to the shoulder of the sound, and could not, without force, be carried further. Thus was demonstrated not only the reduction of the uterus, but that the organ was accurately measured, and found scarcely, if at all, enlarged. The speculum and sound were now withdrawn, the patient carefully removed to the bed, and the bougie retained in place by the hand, to prevent reïnversion. Meanwhile, stimulants were given to sustain the patient, and ergot in such doses as were deemed likely to excite the tonic contraction of the uterus. The patient soon recovered from the effects of the chloroform, and expressed herself as feeling quite as comfortable as before the operation. The patient suffered but little during the operation. The discharge of blood was slight, and when the effects of the chloroform had passed off, and she had taken a little brandy and water, she expressed herself as feeling comfortable. Pulse not sensibly changed in quality, and numbering the same as before the operation.

Drs. Robinson and Batten remained with the patient during the succeeding night, alternately maintaining the bougie in the uterus, supporting it gently, and rendering such other attention as the patient required. Continuing the pressure upon the fundus of the uterus was, perhaps, unnecessary; but it was thought safe not to withdraw the bougie until the next day.

March 13, Dr. Robinson writes: "The patient is quite comfortable; pulse 108. Free from pain. Withdrew the bougie this morning, and found the os uteri embracing it pretty firmly."

Tonics, with nutritious diet, were continued, and quietude in the horizontal position enjoined.

On the 15th, he writes: "The patient is quite comfortable this morning. Made a digital examination, and found the uterus perfectly *in situ*, and mouth well contracted. Has some appetite. Pulse 100."

March 28, he again writes: "She is improving; has been much annoyed by neuralgia and sickness of stomach, but both are giving way, as is the leucorrhœal discharge. The treatment has been sustaining (porter, wine, quinine, iron, &c.,) with nutritious diet." In conclusion, Dr. R. adds: "She will get well, and I feel gratified in the success of the effort of restoration, not only on account of the patient being relieved of a loathsome malady, but also that I have been instrumental in contributing to the professional reputation of one for whom I feel a deep friendship,—as your success in this case will relieve obstetrical surgery from the opprobrium which has hitherto been attached to it in cases of chronic inversion of the uterus."

In reply to a letter of inquiry from me, Dr. Robinson writes, April 22d: "I visited her yesterday and found her very cheerful and able to sit at the table and take tea with us. Her final recovery is now beyond all doubt. In short, the operation has been as successful as its most sanguine friends could desire. Her convalescence has been protracted; slow, perhaps, but when we take into account the great prostration from the long continuance of the malady, and the exhausting hæmorrhages and leucorrhœal discharges to which she had been subjected, the only wonder is that she recovers at all."

I am aware that the history of this case may, to many, seem to be unnecessarily minute; but I have desired, at the risk of being thought tedious, to establish, so far as one case can do, the practicability of the operation of reposition at so late a period after inversion. In order to show the importance of having every step in the procedure as well as its result, and the condition of the organs, when it was undertaken, fully proved, it may be well to cite the language of Dr. Meigs, who doubtless speaks the sentiment of the profession upon the subject, relative to a case of similar duration.*

"It was inverted at the time of her confinement, six months ago. Mrs. Lucina inverted it by pulling at the cord before the placenta was detached, and either did, or did not know what she had done. The hæmorrhage was terrible. The woman ceased to bleed, and did not die, because she fainted so badly, that the vascular injection by the heart was too feeble to kill her by hæmorrhage. She slowly recovered in a measure, but bleeds still upon the smallest excess of exercise or labor.

"Well, now, my young friend, you have made your diagnostic. What are you to do for your patient? Will you reposit or reinstate this womb? You can't. You might as well try to invert one of the non-gravid uteri on my lecture-room table as to reposit this one. The time is gone by. You have no art or skill nor no power equal to the performance of such a miracle of surgery as that."

It may, perhrps, be contended that in the instance of Mrs. Miller the inversion was incomplete until the administration of the aloetic cathartic, when the "pear-shaped tumor first appeared between her thighs accompanied with pains like those of labor and terrible hæmorrhage." This may be true, although the flowing after the delivery of the placenta was so great as to produce "faintings which continued for two days, requiring the constant use of stimulants," showing that incomplete inversion was probably present. Grant, then, that this incomplete inversion existed from the 22d September, the day of delivery, until the 10th of October, eighteen days after, when she took the purge, and when, by her efforts at stool, the inversion was completed. Concede that the os remained dilated, and that the body and neck did not during that period undergo any diminution in size, there is yet more than five calendar months remaining between the 10th of October and the 12th

* Letters to his Class, by Chas. D. Meigs, M. D., pages 231 and 232.

of March—ample time for the complete involution of the uterus before she came under my care. Besides, the measurements of the uterus, accurately taken before and after reduction, show that it was not larger than the standard dimensions of this organ in the multipara, when diminished to its smallest size in the child-bearing period.

Dr. West, in his recent treatise on uterine disease, says that, "unless checked by inflammation, the process of involution, by which the womb is restored to the size and condition which it presented before pregnancy, is *complete in a few weeks*." Nearly all authorities now, I suspect, agree that, when not interrupted by disease, it does not require more than three months completely to effect this change. Here, then, we have the uterus nearly six months after delivery, and more than five after all doubt of the existence of complete inversion, presenting all the appearances, upon careful examination of having undergone complete involution, yielding to the long-continued manipulations, and completely restored by a single effort.

We are all aware that the os and neck of the uterus may, by sponge tents and other mechanical contrivances, be widely dilated when the womb is in its natural position and of ordinary size. This dilation is frequently made by those who are extensively engaged in the treatment of uterine affections for the purpose of perfecting diagnosis or instituting treatment. Of the susceptibility of these parts to be thus dilated, and that, too, without much risk of injury to the tissues involved, or the general health, no one at all familiar with the subject will deny. What is the relation of parts in inversio uteri? Is not the vaginal sheath, which, in the normal arrangement of parts, was attached to the outside of the uterus, now, in the inverted state, firmly attached *within* the cavity of the canal of the neck of the uterus just at its orifice? The vaginal canal is securely attached at its lower extremity at or near the outlet of the pelvis, and whilst it is very elastic, is less yielding in its longitudinal than transverse diameter, and not easily lacerated or detached from its connections, unless irregularly pressed as by a pointed instrument. Force or pressure applied to the fundus of the inverted uterus, is resisted by the upper extremity of the vagina, which is now fastened upon the inside of the neck. The lower extremity of the vagina being firmly attached, cannot yield, and the inevitable result must be the pulling open the mouth of the uterus, unless the tissues are lacerated before that part dilates. Can there be any greater difficulty or danger in *pulling* open this outlet than in *pressing* it open, if performed with the same gentleness?

The uterus and vagina in complete inversion represent a continuous bag or sac, doubled or reflected upon itself, with the open extremity of the sac securely fixed. Pressure upon the closed end of the bag will, it is plainly perceived, under such circumstances, result in straightening the bag by completely turning it the other side out. So with the parts concerned in inversion. Pressure upon the fundus, if well directed, pulls open, first its mouth, then its neck, and finally, if persevered in, doubles the body upon itself also, and carries the fundus through the os and neck and body to its normal position.

Does any uterine pathologist believe it would be impossible safely to dilate the os and bring down the fundus of the uterus—completely inverting the organ—if carefully and perseveringly undertaken? If affirmatively answered, then why may we not pull open the neck by means of the vagina in the same gentle manner as we would press it open when in a normal position, and thus carry the fundus up through it by means of pressure upon that part when it is in a downward direction? Perhaps I may be too sanguine, but I am inclined to believe that well-directed pressure upon the fundus, if continued long enough, will in all cases, where there are no adhesions, result in restoration or reposition, no matter how much time may have elapsed since inversion occurred.

The case of Mrs. M. may indeed be said to establish this point so far as it can be established by a single instance. I am familiar with a case of chronic inversion which occurred sixteen years since, and is described in the article on inversion by Dr. Hunt, already referred to in this paper, and the most careful examination does not enable me to detect any obstacles to its reduction more formidable than had to be overcome in the instance just related. Her general health and her ability to withstand the shock of an operation are greatly superior to the condition of Mrs. Miller. Indeed, I do not despair of inducing this patient to consent to a trial for reduction, which I should undertake with far greater confidence of success than I did the one described above.

When the tissues will not yield to a single effort, some apparatus can easily be adjusted to the fundus of the uterus and retained there, keeping up pressure for several hours or days if necessary. In anticipation of failure to reduce the uterus by a single manipulation, I had provided myself with instruments with which pressure could be continued for a long period without removal from the vagina. Success in the treatment of chronic inversion would be less sanguinely predicted were not the result in this case a mere confirmation of previous opinions formed after much reflection upon the subject. It will be recollected that at the time the inversion of eight days was reduced, the hope was expressed that success would attend the effort of restoration in many cases at a period much later than most writers have heretofore advised the effort to be made.

In relation to the manner in which reduction was effected, it may be well to say one word. There can be no doubt that the os first commenced to yield and pressed down upon the intra-vaginal hand, which, it will be recollected, inclosed the entire uterus and the upper extremity of the bougie and kept them in contact. This part gradually dilated and passed down upon and over the neck, which in turn dilated and doubled down upon itself. The fundus did not perceptibly dimple, or was not reflected upon itself during the operation. The organ was too firm and the cavity too small for any depression to be made upon the walls of the fundus. In recent cases, on the contrary, judging from the one restored within an hour after delivery, and even in that of eight days' standing, it seems to be returned by doubling in or dimpling the fundus, and using it as a wedge to dilate the neck and os. In recent cases much assistance may be rendered by opposing the upper extremity of the uterine tumor with the left hand placed over the hypogastrium. In the chronic case, although the patient was greatly emaciated, the upper extremity of the uterus was too small and obscure to permit counter support to be made available in the operation.

That the administration of chloroform lessens the shock to the nervous system occasioned by the operation, seems highly probable; but it is by no means certain that it renders the reduction any more easily accomplished. Nor am I able to arrive at any satisfactory conclusion relative to the influence exerted by the local application of belladonna.

Buffalo, April 25, 1858.—*Am. Journ. Med. Sciences.*

## Figures and Tables

**Fig. 1. f1:**
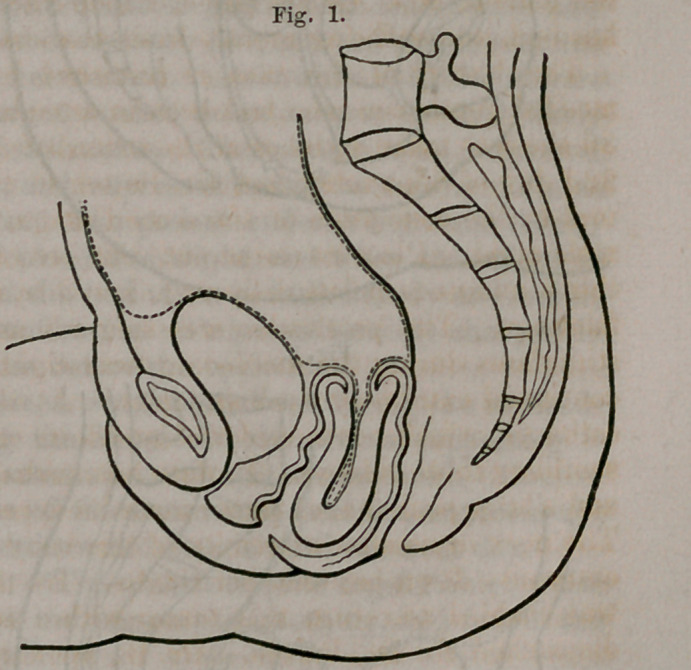


**Fig. 2. f2:**
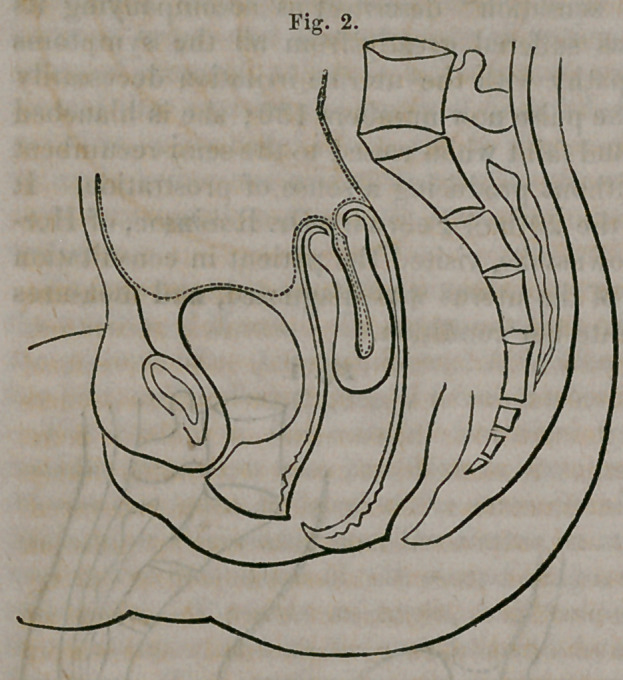


**Fig. 3. f3:**
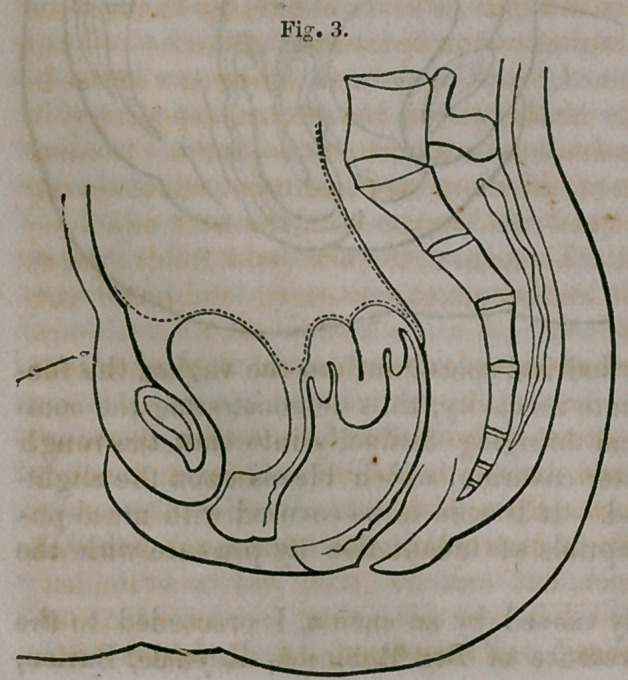


**Fig. 4. f4:**